# Risk of ischaemic heart disease and acute myocardial infarction in a Spanish population: observational prospective study in a primary-care setting

**DOI:** 10.1186/1471-2458-6-38

**Published:** 2006-02-17

**Authors:** Alejandro Marín, María José Medrano, José González, Héctor Pintado, Vicente Compaired, Mario Bárcena, María Victoria Fustero, Javier Tisaire, José M Cucalón, Aurelio Martín, Raquel Boix, Francisco Hernansanz, José Bueno

**Affiliations:** 1 Department of General Medicine, San José Norte Health Centre, Zaragoza, Spain; 2 National Centre for Epidemiology, Carlos III Institute of Public Health, Madrid, Spain; 3 Department of General Medicine, Las Fuentes Norte Health Centre, Zaragoza, Spain; 4 Department of General Medicine, Cariñena Health Centre, Zaragoza, Spain; 5 Department of General Medicine Illueca Health Centre, Zaragoza, Spain; 6 Arterial Hypertension Research Foundation, Zaragoza, Spain; 7 Department of Internal Medicine, University Clinical Teaching Hospital, Zaragoza, Spain

## Abstract

**Background:**

Ischaemic heart disease is a global priority of health-care policy, because of its social repercussions and its impact on the health-care system. Yet there is little information on coronary morbidity in Spain and on the effect of the principal risk factors on risk of coronary heart disease. The objective of this study is to describe the epidemiology of coronary disease (incidence, mortality and its association with cardiovascular risk factors) using the information gathered by primary care practitioners on cardiovascular health of their population.

**Methods:**

A prospective study was designed. Eight primary-care centres participated, each contributing to the constitution of the cohort with the entire population covered by the centre. A total of 6124 men and women aged over 25 years and free of cardiovascular disease agreed to participate and were thus enrolled and followed-up, with all fatal and non-fatal coronary disease episodes being registered during a 5-year period. Repeated measurements were collected on smoking, blood pressure, weight and height, serum total cholesterol, high-density and low-density lipoproteins and fasting glucose. Rates were calculated for acute myocardial infarction and ischaemic heart disease. Associations between cardiovascular risk factors and coronary disease-free survival were evaluated using Kaplan-Meier and Cox regression analyses.

**Results:**

Mean age at recruitment was 51.6 ± 15, with 24% of patients being over 65. At baseline, 74% of patients were overweight, serum cholesterol over 240 was present in 35% of patients, arterial hypertension in 37%, and basal glucose over 126 in 11%. Thirty-four percent of men and 13% of women were current smokers. During follow-up, 155 first episodes of coronary disease were detected, which yielded age-adjusted rates of 362 and 191 per 100,000 person-years in men and women respectively. Disease-free survival was associated with all risk factors in univariate analyses. After multivariate adjustments, age, male gender, smoking, high total cholesterol, high HDL/LDL ratio, diabetes and overweight remained strongly associated with risk. Relative risks for hypertension in women and for diabetes in men did not reach statistical significance.

**Conclusion:**

Despite high prevalence of vascular risk factors, incidence rates were lower than those reported for other countries and other periods, but similar to those reported in the few population-based studies in Spain. Effect measures of vascular risk factors were mainly as reported worldwide and support the hypothesis that protective factors not considered in this study must exist as to explain low rates. This study shows the feasibility of conducting epidemiological cohort studies in primary-care settings.

## Background

Ischaemic heart disease (IHD) is an extremely frequent cause of hospitalisation and death in Spain. This has made it a priority of health-care policy, not only because of its social repercussions, but also because of its impact on the health-care system [1]. Furthermore, incidence studies conducted in Spain indicate that IHD is a frequent disease, with rates in the order of 200 and 50 new myocardial infarctions p.a. per 100,000 men and 100,000 women respectively [2-8]. At an international level, however, these rates are among the lowest in the world, notwithstanding the fact that frequency of exposure to cardiovascular risk factors is no less among Spaniards than it is in other countries [9,10]. This phenomenon, known as the French paradox, is also seen in other populations in the Mediterranean area and has been linked to the possible existence of protective factors linked to the Mediterranean diet [11-13]. Yet there is little information on the effect of the principal risk factors on risk of coronary heart disease in populations with low rates of IHD morbidity and mortality [14,15]. Indeed, it has been postulated that the magnitude of the effect had by the principal cardiovascular risk factors on such populations might be different to that had on populations registering high rates [16].

In addition, the coronary morbidity information available for Spain and for Europe in general, is sparse, only marginally comparable and subject to certain limitations [8,15]. A recent review, ordered by the European Commission as part of the Health Monitoring Programme, concluded that cardiovascular disease morbidity data are rarely available in the different countries, and when available, are very rarely comparable [17]. Moreover, longitudinal studies undertaken in Europe are already out of date and their results on incidence cannot be extrapolated to the present [15,18]. In Spain, neither the MONICA-Catalonia study, nor the registers constituting the IBERICA study, allow for the risk associated with exposure to risk factors to be estimated [2-5]. Moreover, both studies solely collect information on the incidence of acute myocardial infarction and not on other forms of cardiac ischaemia. The Manresa cohort study was confined to men from a very restricted work setting, whereas, to date, the DRECE study has not reported results on coronary incidence by age and sex [6,7].

Primary-care settings, mostly in those health systems that as the Spanish one cover the entire population, could be a good alternative to population-based registries to conduct epidemiological studies, as general practitioners are fully qualified and capable for detecting both cardiovascular risk of their patients and coronary events and deaths, and moreover this information is routinely recorded in primary-care clinical practice. This study sought: to describe coronary disease morbidity and mortality across 5 years of follow-up in a cohort of persons attended in a primary-care setting; and to analyse the association between such morbidity and mortality, and subjects' serum cholesterol levels, blood pressure, glucaemia, tobacco use and overweight.

## Methods

Eight primary-care centres in the Province of Zaragoza participated in the study, each contributing to the constitution of the cohort with the entire population assigned by the Spanish National Health Service to each of the participating doctors. In May 1994, a closed cohort of 6262 men and women over the age of 25 years was thus formed. The patients were base-line interviewed and explored after obtaining their informed consent, at the time of requesting some type of service, regardless of the reason for their medical visits. All patients who presented with history of ischaemic heart disease or cerebrovascular accident were excluded. Patients were subsequently followed up on a consultation-on-demand basis until May 1999; during this 5 year period all episodes of fatal and non-fatal ischaemic heart disease were systematically searched and duly registered. On conclusion of the study, all patients who had not attended the primary-care centre in the preceding 9 months were contacted by telephone to identify any possible non-reported events.

An ischaemic cardiac event (codes 410 to 414, Ninth Revision of the International Classification of Diseases: ICD-9) was defined as any event diagnosed as acute myocardial infarction, non-Q wave myocardial infarction, unstable angina, angina or coronary angioplasty by the cardiologists' or reference hospitals' reports, or death certificates in the case of sudden deaths. From these, only those cases that fulfilled MONICA diagnostic criteria were classified as AMI (definite or probable). No attempt was made to recode cases according to the new definition of acute coronary syndromes, because the information that would have been needed to do so was not gathered at the time the study was conducted. All events were validated against clinical records. For this study only incident first-ever events were considered; in case of multiple or recurrent events only the first one was considered.

A total of 138 individuals (2.2%) who could not be followed up after enrolment, were deemed to be lost to follow-up. Consequently, the final cohort numbered 6124 persons (3393 women and 2731 men).

At baseline (date of enrolment) and across follow-up, the following information was collected on each patient: sex, age, smoking habit, systolic and diastolic blood pressure, weight and height, total cholesterol, high-density lipoprotein cholesterol (HDL-c), low-density lipoprotein cholesterol (LDL-c), triglyceride and glucose. To standardise the recording of data, the eight researchers used the "HIPÓCRATES" clinical-history-management software programme as supplied by the Spanish General Medicine Society (SEMG).

Biochemical methods were those routinely used in Spanish primary-care system. Blood was extracted after a 10–12 hour fast. Extracted specimens were immediately refrigerated and stored until delivery that same day to the laboratory. Total cholesterol and HDL-cholesterol concentrations were determined by the cholesterol oxidase: p-aminophenazone (CHOD-PAP) method (enzymatic colorimetric test), after separation by precipitation and centrifugation of kilomicrons, VLDL and LDL. LDL concentration was calculated using Friedewald's formula [19]. Determination of glucaemia was performed using the hexokinase-based GLUCO-QUANT method.

Blood pressure was measured twice with the subject seated and at rest, using a mercury sphygmomanometer, as per the Hypertension Detection and Follow-up Program Guidelines [20]. Smoking habit was evaluated by interviewing subjects as to their present and past history of tobacco use. Condition of previous ischaemic event was likewise assessed. We defined: a smoker as any individual who had regularly smoked a minimum of 1 cigarette per day up to two years prior to the study termination date; and a non-smoker as any patient that had never regularly smoked 1 or more cigarettes per day, or had done so more than 2 years prior to the study termination date.

### Statistical analysis

Frequency of coronary events and coronary and all-cause mortality were calculated by means of incidence and mortality rates per person-years of observation, with the follow-up period defined as the time elapsed from the start of the study to the date of appearance of the event or death, where applicable, or alternatively, from the start of the study to the study termination date (May 1999); in the case of mortality rates, the time was deemed to run until the date of death or termination of the study. Rates were adjusted for age, taking the age distribution of the Standard European population as reference.

Survival analysis was used to describe and analyse the experience of coronary morbidity in the cohort, with the Kaplan-Meier method for the univariate analyses and Cox proportional risk regression for the multivariate analysis. In both cases, the association between IHD-free survival and cardiovascular risk factors was evaluated in accordance with pre-established dichotomous categories of risk factors. For each such factor (systolic and diastolic blood pressure, total cholesterol, LDL- and HDL-cholesterol, glucaemia, weight), we took the mean value of the repeated measures obtained across follow-up. The criterion used to define arterial hypertension (AHT) was the Sixth Report of the Joint National Committee, which defined hypertension as systolic blood pressure (SBP) ≥ 140 mm Hg and/or diastolic blood pressure (DBP) ≥ 90 mm Hg [21]. Hypercholesterolaemia was defined as total cholesterol ≥ 240 mg/dl as per NCEP (ATP II) criteria [22]. The effect of the LDL and HDL lipoprotein level was jointly assessed by reference to the atherogenic index (LDL/HDL ratio); for the purpose of this index, a cut-off of 3 was used to define the categories. The diagnostic criterion for definition of diabetes mellitus was fasting plasma glucose ≥ 126 mg/dl, as per the ADA recommendations [23]. Overweight was defined as a Body Mass Index (BMI; weight, expressed in kilograms, divided by the square of the height, expressed in metres) > = 25. Treated patients were classified as exposed when the mean of the repeated measures was above the cut-off values; this way, not controlled patients remained as exposed and controlled patients were classified either as exposed or not exposed depending on the degree of effective control.

To avoid overadjustment, overweight was excluded from the Cox model in assessing the effect of diabetes and AHT, and LDL/HDL ratio in measurement of the effect of hypercholesterolemia, since they share the etiological pathway. To avoid potential bias and power shortages due to deletion of cases in complete-case analysis, missing data in covariates were replaced by multiple imputation, with age, sex and coronary disease outcome taken as predictors [24]. If missing data in a variable were more than 30%, these were not imputed and the variable considered as measured only in a subgroup of the cohort. This occurred in BMI and LDL/HDL ratio variables.

The study complies with the World Medical Association Declaration of Helsinki. Due to the non-experimental nature of the research, the study protocol did not need to be submitted for consideration and approval to an ethical review committee.

## Results

The general characteristics of the cohort, comprising 6124 patients (44.6% men, 55.4% women) are described in Table 1. A more detailed description has been previously published [25]. The age range was 25–99 years (mean ± standard deviation 50.4 ± 15 and 52.8 ± 16 in men and women respectively, p = 0.000), with 24% of patients being over 65 and 9% over 75 years of age. The prevalence of the cardiovascular risk factors considered was as follows: 34% of men and 13% of women were current smokers (p = 0.00); 76% and 73% respectively were overweight (p = 0.02); and 33 and 37% respectively were hypercholesterolaemic (p = 0.03). Frequency of arterial hypertension (38% in men, 36% in women) and diabetes mellitus (11%) showed no statistically significant differences between the sexes.

**Figure 1:**
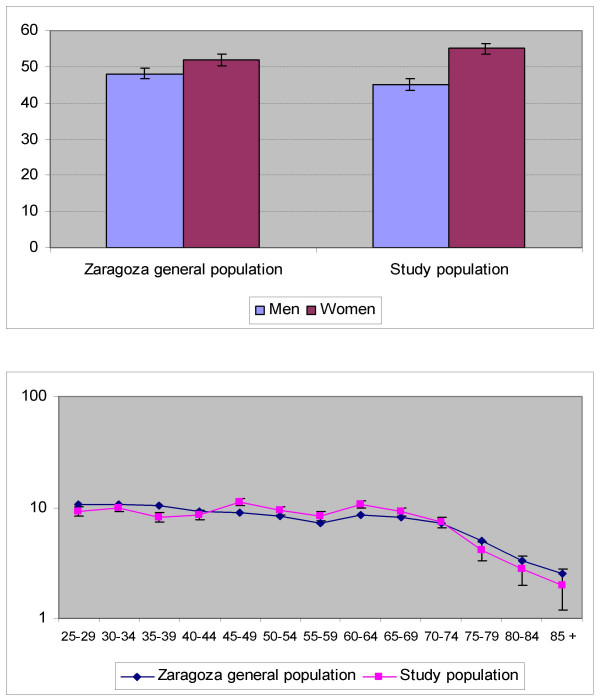
Age and gender distribution of the study population and of the Zaragoza general population.

The age and gender distributions of the study cohort vis-à-vis those of the Zaragoza general population obtained from official demographic statistics are depicted in Figure 1. These distributions in the study population overlaps that of the general population, with the differences of female (55% vs. 52%) and the 35–64 age group (56% vs. 52%) being slightly over represented in the cohort.

Over the 5 years of study, the 6124 patients included in the cohort furnished 30,386 person-years of observation (mean ± standard deviation 4.96 ± 0.03 years), with 159 patients (0.02%) with incomplete follow-up due to competing mortality. A hundred and fifty-five first episodes of all forms of ischaemic heart disease were detected (fatal and non-fatal) (Table 2). The crude incidence rates per 100,000 person-years were 650 in men and 403 in women; the age-adjusted rates remained higher for men than for women, with figures of 362 and 191 new cases per 100,000 person-years respectively, and increased with age in both sexes. This incidence pattern -namely, increasing with age, and higher in men than in women- was maintained for acute myocardial infarction alone.

Twenty-one of the 155 episodes of ischaemic heart disease resulted in death, yielding an age-adjusted IHD mortality rate of 42.5 and 25.8 per 100,000 person-years, for men and women respectively (Table 2). All-cause mortality in this cohort was 5.92 per 1000 person-years (adjusted rate of 3.15 per 1000).

**Figure 2:**
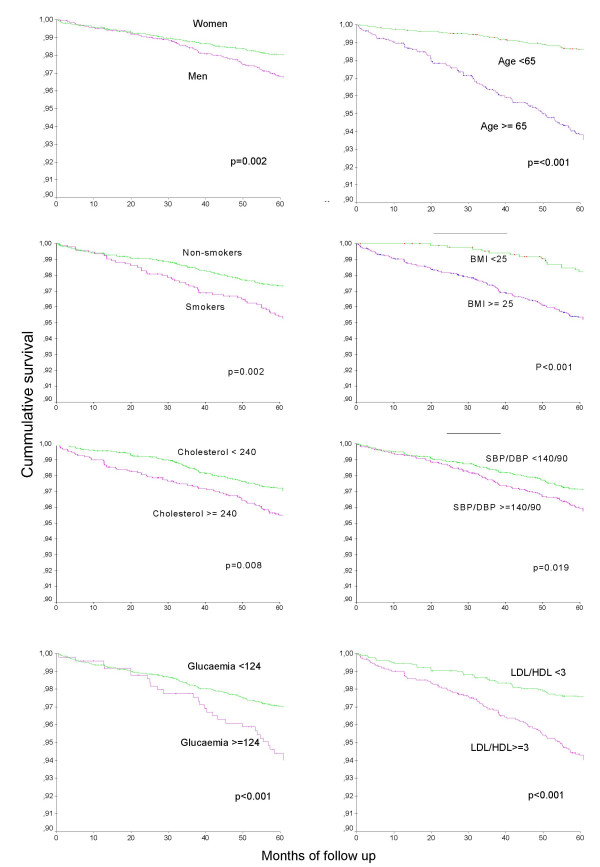
Ischaemic heart disease-free survival by category of vascular risk factors.

All the risk factors considered displayed an statistically significant association with incidence of ischaemic heart disease in the univariate analysis (Figure 2, table 3), the strongest associations being observed for age and overweight. After multivariate adjustments, age, male gender, smoking habit, hypercholesterolaemia and the LDL/HDL ratio remained strongly and consistently associated with risk of suffering ischaemic heart disease after five years of follow-up (Table 4). The positive, significant effect of age, as well as those of cholesterol, LDL/HDL ratio and BMI on risk remained significant when they were taken in a continuous scale. The presence of hypertension, that showed a 24% increase in risk that did not reach statistical significance in the analysis for the hole cohort, was statistically associated with disease in men but not in women. Positive and high relative risks for overweight an smoking in women were almost statistically significant. Lastly, diabetes proved to be associated with disease in women but not in men.

There were only three cases of sudden death, two of them presenting symptoms of severe neurological deficit and no chest pain. These contributed to follow up time until the date of death, which was recorded as due to probable stroke.

The one single case of sudden death with AMI compatible symptoms was considered as probable AMI, following MONICA criteria. The inclusion or exclusion of this case of the analysis did not modify the results.

## Discussion

Knowledge of the magnitude of IHD risk, along with identification of the factors responsible and populations at greatest risk, are fundamental for the planning and assessment of preventive and health-care strategies and identification of specific excess-risk scenarios. There is a dearth of such information in Spain. This study sought to describe the epidemiology of ischaemic heart disease and acute myocardial infarction in a cohort drawn from the population attending primary-care centres.

Epidemiological studies conducted in a primary care setting can be subject to several limitations, that must be taken into account [26]. Firstly, this cohort cannot be regarded as representative of the general population but only of those who use primary-care services, although it should be stressed that the Spanish National Health Service covers the entire population and that, according to the Spanish National Health Survey, 80% of the Spanish adult population attends such health centres at least once a year [27]. In this study, the age and sex distribution of the cohort was very similar to that of the general population, but substantial differences in frequentation to primary care by age and sex have been well described in many studies, so it is likely that detection and exposure classification biases could be present; to minimize this potential biases the study protocol included systematic search of events and exposures during follow-up and at the end of the study period. This systematic approach explains the very low percentage of patients lost for follow-up. Anyway, we must further remark that the study population is not a random sample of general population, so any generalization of the results should be cautious. Furthermore, the cohort is not a sample but the entire population covered by the participant health centres but these were not randomly sampled, this meaning that the results are applicable only to the study population, and of course this population is not representative of other populations.

Clinical trials have demonstrated that effective medical control of vascular risk factors reduces risk, so classifying treated patients as exposed (as has traditionally been done) could have biased the results, mostly when the study was conducted in a medical setting. In the same manner, including treated patients as not exposed would have also introduce a bias because effective control is not always achieved. This potential exposure misclassification derived from medical treatment and control has been approached by taking the mean of repeated measures for arterial pressure, serum cholesterol and glucose, so treated patients were therefore classified as exposed when the mean of the repeated measures was above the cut-off values; this way, not controlled patients remained as exposed and controlled patients were classified either as exposed / not exposed depending on the degree of effective control.

In the same way, multiple imputation of missing data was used to prevent biases derived from the aggregation of missing data in different variables when considering them jointly in multivariate analyses [24].

The age-adjusted acute myocardial infarction incidence rates registered in this study are similar to those of the IBERICA study for the general population registers of 7 Spanish regions as a whole, the MONICA-Catalonia, the REGICOR and the IBERICA-Murcia studies [2,4,5,28]. Nevertheless, it should be borne in mind here that our study targeted an older population, i.e., whereas in the above studies the study population was aged 25–75 years, in our cohort 10% of women and 5% of men were over 75 years old. Still, when this age breakdown was taken into account, the incidence rates in our cohort were almost the same as those found in the general population, despite the fact that this was a medical-settled study. The likelihood of information on events in the cohort being lost is practically non-existent, since the researchers were the designated general practitioners of the patients enrolled, and therefore responsible for their medical follow-up and prescription of drugs in the case of cardiovascular episodes. At all events, on termination of the study all patients who had not attended the primary-care centre in the preceding 9 months were contacted by telephone.

In Spain, only two other cohort studies have been conducted to date, aimed at measuring and analysing the risk of IHD, namely the Manresa and DRECE studies, though their results are not entirely comparable vis-à-vis our study or even each other, due to differences in methodology and design [6,7]. Taking the above limitations on comparability into account, it can be concluded that the incidence rates registered in the present study do not diverge from those published in Spain until now, and that any differences are attributable to design. This incidence is very high compared to Spanish incidence rates for other chronic diseases, and indeed exceeds the joint incidence of colon, rectal, lung and breast cancer combined [29]. In the international context, the myocardial infarction incidence rate in this population is very low compared to those reported for non-Spanish populations in the MONICA study [2] and under-65 mortality is lower than that reported in cohorts included in the SCORE project, though these cohorts were studied 20–30 years ago [15].

IHD incidence in this cohort should be interpreted in the light of its level of cardiovascular risk. Compared to the general Spanish population, this cohort registered comparable prevalences of arterial hypertension and diabetes, slightly higher prevalences of overweight and hypercholesterolaemia, and a lower prevalence of tobacco use. The differences in risk profile in studies targeting persons attending primary-care centres is known and has been previously reported [9]. However, with the sole exception of the low frequency of smoking (34% and 13% among men and women respectively versus 40% and 25% in the general population), the differences in this study are not unduly pronounced, something that would lead one to expect IHD incidence rates to be within the range of those reported by population-based studies in Spain, as is indeed the case.

Analysis of the association between cardiovascular risk factors and risk of ischaemic heart disease yields results in line with what was expected, with age, smoking, elevated LDL-cholesterol, hypertension, diabetes and overweight emerging as linked to the risk of suffering an event. This is the first ever cohort study in Spain to show these associations, since previous studies were unable to rule out the null hypothesis for overweight, dyslipaemia or diabetes [6,7]. Insofar as arterial hypertension is concerned, the absence of statistical significance in the association among women may be reflecting a better medical control of AHT than among men. Information on treatment and effective control of AHT was not collected in this study, and we believe that further research is needed to identify possible gender differences in AHT awareness, treatment and control in Spain.

Lastly, this study shows the feasibility of conducting epidemiological studies in primary-care settings. Furthermore, the opportunities offered by the primary health care setting were not utilised fully in this study, i.e. treatment information was not recorded when it would have been feasible and desirable. Particular strenghths were found, as the minimization of follow-up and event losses or diagnostic misclassifications. Potential weaknesses, as selection biases due to demographic differences of primary-care frecuentation can been also minimized by an adequate, systematic approach. This systematic approach should also be used to prevent the miss of data, a possibility always present in large cohort studies, moreover with the high work load of primary-care doctors.

## Conclusion

To sum up, IHD incidence and mortality rates were lower than those reported for other countries and other periods, despite a high prevalence of cardiovascular risk factors, thus confirming similar results reported in other cohort and population-based studies in Spain. Effect measures of vascular risk factors were mainly as reported worldwide and support the hypothesis that protective factors not considered in this study must exist as to explain low rates.

## Competing interests

The author(s) declare that they have no competing interests.

## Authors' contributions

AM conceived of the study, participated in its design and coordination and helped to draft the manuscript. MJM and RB performed the statistical analysis and interpretation of data and prepared the draft manuscript. JG, HP, VC, MB, MVF, JT, JMC, AM, FH and JB participated in the design of the study, in patients recruitment, follow-up and data collection, and in critical review of the manuscript. All authors read and approved the final manuscript.

## Pre-publication history

The pre-publication history for this paper can be accessed here:



## Figures and Tables

**Table 1:** General characteristics of the cohort compared to general population.

	**Cohort **(%)		General population *(%)	
**Age**				
25–29	9		11	
30–34	10		11	
35–39	8		10	
40–44	9		9	
45–49	11		9	
50–54	9		8	
55–59	8		7	
60–64	11		9	
65–69	9		8	
70–74	7		7	
75–79	4		5	
80–84	3		3	
85 and over	2		3	
				
**Gender**				
Male	45		48	
Female	55		52	
				
**Vascular risk factors**	**Male**	**Female**	**Male**	**Female**
				
Current smoking	34	13	40,7 (37–44)	24,1 (21–26)
Serum total cholesterol >= 240 mg/dl	33	37	27,3 (17–37)	23,4 (16–31)
Arterial pressure >= 140/90 mmHg	38	36	35,6 (27–44)	31,8 (24–39)
Glucose > = 126 mg/dl	11	11	12,5 (11–14)	7,9 (7–9)
Body mass index>= 25	76	73	66,9 (63–71)	48,3 (41–55)

* Age and sex distribution in general population (province of Zaragoza) obtained from census data. Prevalence and 95% confidence interval (in brackets) of vascular risk factors in Spanish general population obtained from reference 9.

**Table 2:** Incidence and mortality due to Ischaemic Heart Disease (IHD) and Acute Myocardial Infarction (AMI).

**Age in years**	**n**	**Person-years**	**No. IHD events**	**IHD Incidence rate × 10^5^person-years**	**No. AMI events**	**AMI Incidence rate × 10^5^person-years**	**No. IHD deaths**	**IHD Mortality rate × 10^5^person-years**
**MEN**								
25–29	272	1,377.6	1	72.6	1	72.6	0	0.0
30–34	295	1,491.1	1	67.1	1	67.1	0	0.0
35–39	225	1,138.7	1	87.8	1	87.8	0	0.0
40–44	241	1,218.6	1	82.1	1	82.1	0	0.0
45–49	321	1,614.2	5	309.7	1	61.9	0	0.0
50–54	266	1,321.3	8	605.5	2	151.4	0	0.0
55–59	238	1,163.0	13	1,117.8	9	773.9	1	86.0
60–64	286	1,402.8	14	998.0	4	285.2	1	71.3
65–69	242	1,153.7	20	1,733.6	12	1,040.2	5	433.4
70–74	186	880.8	14	1,589.5	7	794.7	0	0.0
75–79	80	394.1	4	1,015.0	2	507.5	1	253.7
80–84	57	264.6	6	2,267.4	4	1,511.6	3	1,133.7
85 and over	22	108.8	0	0.0	0	0.0	0	0.0
25 and over (crude)	2,731	13,529.2	88	650.4	45	332.6	11	81.3
25 and over (adjusted)				362.7		188.0		42.5
								
25–74 (adjusted)	2,572	12,761.7	78	366.7	39	183.4	7	32.9
**35–64 (adjusted)**	1,872	9,349.7	43	211.6	19	93.5	2	9.8
								
**WOMEN**								
25–29	293	1,485.4	0	0.0	0	0.0	0	0.0
30–34	311	1,576.6	0	0.0	0	0.0	0	0.0
35–39	271	1,373.8	0	0.0	0	0.0	0	0.0
40–44	283	1,434.7	0	0.0	0	0.0	0	0.0
45–49	360	1,815.6	2	110.2	0	0.0	0	0.0
50–54	304	1,533.1	2	130.5	2	130.5	0	0.0
55–59	276	1,375.6	7	508.9	2	145.4	0	0.0
60–64	362	1,801.8	8	444.0	1	55.5	0	0.0
65–69	314	1,506.6	17	1,194.7	7	464.6	3	199.1
70–74	266	1,314.1	8	608.8	0	0.0	0	0.0
75–79	172	806.1	13	1,612.7	3	372.2	3	372.2
80–84	114	534.9	7	1,308.8	3	560.9	2	373.9
85 and over	67	299.3	3	1,002.2	3	1,002.2	2	668.2
25 and over (crude)	3,393	16,857.5	67	403.4	21	124.6	10	59.3
25 and over (adjusted)				191.0		62.3		25.8
								
25–74 (adjusted	3,040	15,217.2	45	177.4	12	47.3	3	11.8
**35–64 (adjusted)**	2,167	10,911.2	19	80.1	5	21.1	0	0

**Table 3:** Ischaemic heart disease incidence by category of exposure to cardiovascular risk factors.

		BOTH SEXES				MEN				WOMEN			
		No. patients	No. events	Cumulative incidence rate (%)	p	No. patients	No. events	Cumulative incidence rate (%)	p	No. patients	No. events	Cumulative incidence rate (%)	p

AGE	<65	4,671	64	1.4		2,169	44	2.0		2,502	20	0.8	
	>= 65	1,453	91	6.3	<0.001	562	44	7.8	<0.001	891	47	5.3	<0.001
													
CURRENT SMOKING	No	3,361	90	2.7		1,230	38	3.1		2,131	52	2.4	
	Yes	959	44	4.6	0.002	642	39	6.1	0.003	317	5	1.6	0.380
													
LDL/HDL RATIO	<3	1,781	43	2.4		572	21	3.7		1,209	22	1.8	
	> = 3	1,615	95	5.9	<0.001	884	59	6.7	0.016	731	36	4.9	<0.001
													
SERUM TOTAL CHOLESTEROL (mg/dl)	<240	2,793	81	2.9		1,207	49	4.1		1,586	32	2.0	
	> = 240	1,513	67	4.4	0.008	597	35	5.9	0.091	916	32	3.5	0.022
													
ARTERIAL PRESSURE (mmHg)	<140/90	2,818	81	2.9		1,161	45	3.9		1,657	36	2.2	
	> = 140/90	1,655	69	4.2	0.019	723	41	5.7	0.086	932	28	3.0	0.188
													
GLUCOSE (mg/dl)	<126	3,940	118	3.0		1,635	73	4.5		2,305	45	2.0	
	> = 126	494	29	5.9	0.001	215	11	5.1	0.666	279	18	6.5	<0.001
													
BODY MASS INDEX	<25	857	15	1.8		327	8	2.4		530	7	1.3	
	> = 25	2,468	117	4.7	<0.001	1,054	66	6.3	0.007	1,414	51	3.6	0.008

**Table 4:** Multivariate adjusted effect of cardiovascular risk factors on incidence of ischaemic heart disease.

	BOTH SEXES				MEN				WOMEN			
	RR	IC 95%		p	RR	IC 95%		p	RR	IC 95%		p

MALE GENDER	1.65	1.16	2.34	.005	-	-	-	-	-	-	-	-
AGE >= 65	4.93	3.45	7.06	<0.001	4.04	2.54	6.40	<0.001	6.71	3.72	12.09	<0.001
CURRENT SMOKING	2.60	1.75	3.85	<0.001	2.72	1.73	4.26	<0.001	2.03	.77	5.32	.151
CHOLESTEROL >= 240	1.97	1.42	2.73	<0.001	1.95	1.26	3.00	.003	1.92	1.17	3.14	.010
SBP/DBP >= 140/90	1.24	.88	1.73	.218	1.61	1.04	2.50	.033	.80	.47	1.36	.412
GLUCOSE >= 126	1.52	1.00	2.33	.050	.92	.47	1.78	.798	2.51	1.41	4.45	.002
												
BMI > = 25	2.21	1.28	3.83	.004	2.31	1.10	4.86	.027	2.03	.90	4.59	.089
LDL/HDL RATIO >= 3	2.12	1.40	3.20	<0.001	1.80	1.02	3.16	.042	2.43	1.34	4.40	.003
